# Ultrasound evaluation in the postoperative period for surgical treatment of thoracic infectious and inflammatory diseases

**DOI:** 10.1016/j.clinsp.2024.100452

**Published:** 2024-08-07

**Authors:** Mauro Razuk Filho, Fabio Eiti Nishibe Minamoto, Alessandro Wasum Mariani, Paula Duarte D'Ambrosio, Thiago Potrich Rodrigues, Maria Cristina Chammas, Ricardo Mingarini Terra, Paulo Manuel Pêgo-Fernandes

**Affiliations:** a Instituto do Coracao, Hospital das Clinicas HCFMUSP, Faculdade de Medicina, Universidade de Sao Paulo, Sao Paulo, SP, Brazil; b Instituto de Radiologia, Hospital das Clinicas HCFMUSP, Faculdade de Medicina, Universidade de Sao Paulo, Sao Paulo, SP, Brazil

**Keywords:** Thoracic surgery ultrasonography, Radiography, Thoracic, Thoracic Cavity Thoracic Diseases

## Abstract

**Objective:**

This study aims to evaluate the role of TUSG in the postoperative period and the detection of early complications after surgical treatment, pulmonary resection, or decortication for infectious and inflammatory thoracic diseases, comparing with the standard method (Chest Radiography ‒ CXR).

**Methods:**

Prospective non-randomized self-controlled study. Twenty-one patients over 16 years of age have undergone surgical treatment of inflammatory and infectious lung diseases. These patients were followed up with CXR and TUSG (performed on the 1^st^ and 3^rd^ postoperative days and/or after the chest tube removal).

**Results:**

Both exams demonstrated similar results regarding their ability to safely predict the adequate moment for chest drain removal. TUSG allowed chest drain removal in 30% of cases and CXR in 34%. Statistical analysis demonstrates that both exams have similar capabilities in detecting postoperative changes in the pleural space. However, the authors report that TUSG is statistically more accurate in detecting subcutaneous emphysema than CXR (p = 0.037, Kappa [κ = 0.3068]). The analysis of other parameters showed no statistical difference.

**Conclusion:**

The authors conclude that TUSG in trained hands is equivalent to CXR in searching for postoperative complications regarding the surgical treatment of infectious and inflammatory thoracic diseases and can be used as a complement, and not a substitute, to CXR, when CCT is not feasible, or a more urgent diagnosis is needed.

## Highlights

• TUSG aids post-op care, no radiation.
• CXR limits: 2D, discomfort, radiation.
• Study compares TUSG to CXR.
• TUSG complements CXR, not a replacement.

## Introduction

Thoracic Ultrasonography (TUSG) has been an essential tool in the diagnosis of medical urgencies and emergencies due to its easy handling, portability, and speed. It can be performed at the bedside in any hospital environment.^1^ Despite this, its use is still limited in the postoperative follow-up of thoracic surgery, with Chest Radiography (CXR) being the initial examination and Chest Computed Tomography (CCT) the gold standard for the diagnosis of complications.^2^

CXR is the standard test used in postoperative follow-up in thoracic surgery. Although recent studies are still trying to corroborate the need for routine radiography for these patients, it is well accepted that this diagnostic method has limitations (two-dimensional examination, need for patient mobilization that can cause pain and discomfort, patient exposure to radiation) that may delay adequate patient care.^3^, ^4^, ^5^ The TUSG, as a diagnostic method, is validated in pulmonology and medical emergency scenarios, does not require patient transport, presents real-time imaging, and avoids radiation exposure. In experienced hands, it has a high sensitivity for differentiating between atelectasis, consolidations, pleural effusion, and detecting pneumothorax before and after drain removal. It also allows the evaluation of pneumonia and pulmonary edema before and after interventions.^5^, ^6^, ^7^, ^8^, ^9^

Few studies demonstrate the applicability of TUSG in the postoperative follow-up in thoracic surgery, and most were performed with small groups of patients and directed to post-pulmonary resection care, mainly for the treatment of neoplasms. Nevertheless, these studies show promising results. They indicate that, when performed by an experienced professional who is aware of the clinical context, TUSG can be a low-cost, rapid tool that avoids radiation exposure to identify complications and even chest tube management.^3^^,^^4^^,^^10^^,^^11^ However, to our knowledge, there is no published data regarding the use of the TUSG for the postoperative care of patients with infectious and inflammatory diseases treated by lung resection or pulmonary decortication, which are a special population with a higher tendency of postoperative pleural complications.^12^

Therefore, this study aims to evaluate the role of TUSG in the postoperative period and the detection of early complications after surgical treatment, pulmonary resection or decortication for infectious and inflammatory thoracic diseases, comparing with the standard method (CXR). The authors also endeavored to assess whether TUSG can be used as a predictor for chest tube removal and whether this method effectively detects immediate complications after tube removal.

## Methods

Prospective non-randomized self-controlled case series (single group) approved by the Scientific Committeesity of São Paulo ãand CAPPESQ of the Hospital das Clinicas of the Univeristy of Sao Paulo (CAAE Number 54681221.6.0000.0068). Twenty-one patients over 16 years of age that have undergone surgical treatment of inflammatory and infectious lung diseases through lung resections (segmentectomy and lobectomy) and pleural decortication, open or by Videothoracoscopy (VATS), hospitalized at the tertiary academic hospital (Hospital das Clinicas da Universidade de Sao Paulo, HC-FMUSP)ã, under the care of the Thoracic Surgery Department, were included in the study.

These patients were followed up with CXR (current departmental routine consists of performing CXR in the immediate postoperative period and daily until removal of the chest tube) and TUSG (research protocol). The only exclusion criterion will be the refusal to sign the Free and Informed Consent Form.

**Fig. 1:**
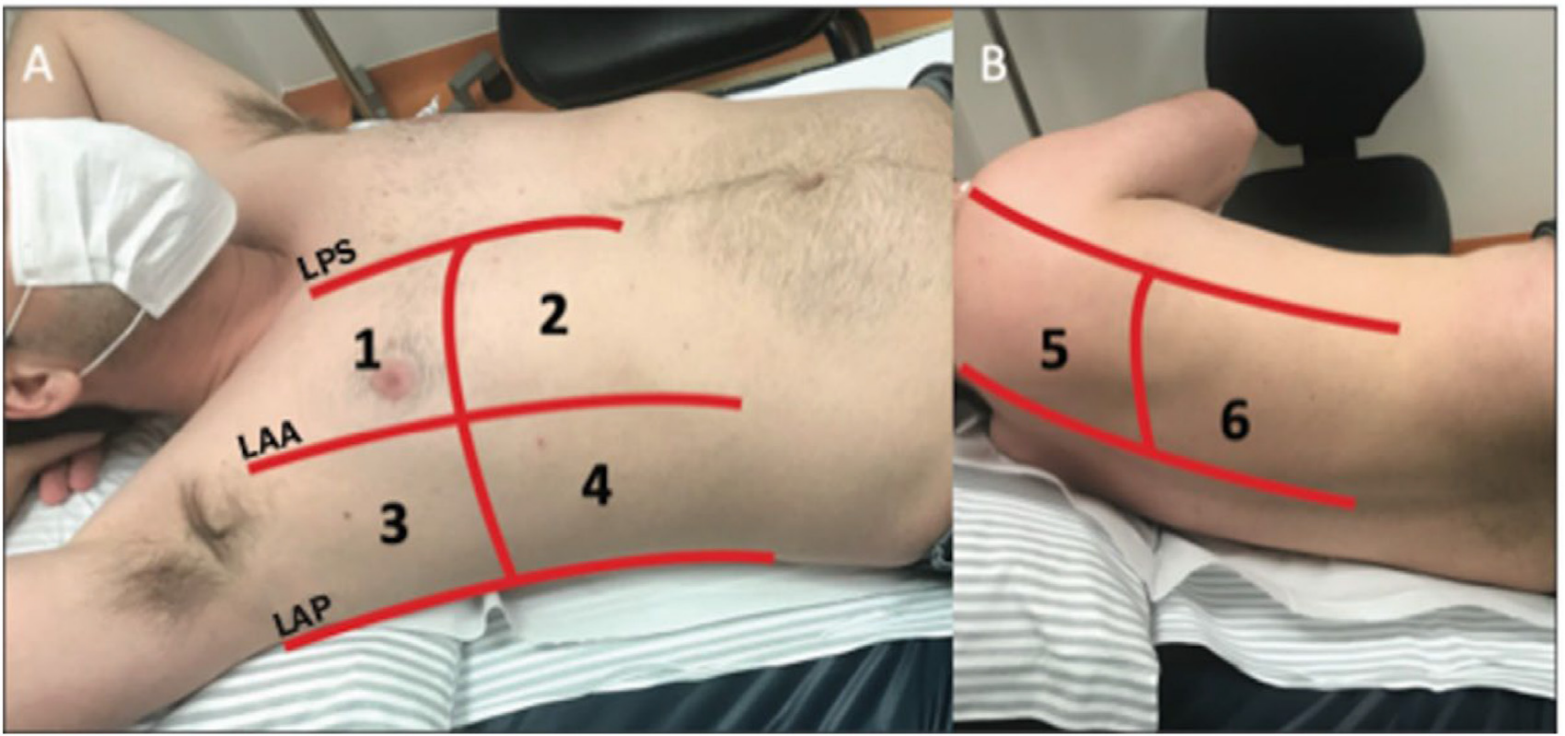
Ultrasound zones of the chest (taken from Oliveira RR, Rodrigues TP, Savoia P, Gomes AC, Chammas MC. Lung ultrasound: an additional tool in COVID-19. Radiol Bras.); (A) Horizontal supine position. (B) Left lateral decubitus. LPS, Parasternal Line; LAA, Anterior Axillary Line; LAP, Posterior Axillary Line. 1, Anterosuperior region; 2, Anteroinferior region; 3, Upper lateral region; 4, Lower lateral region; 5, Posterosuperior region; 6, Posteroinferior region.

The TUSG was performed on the 1^st^ and 3^rd^ postoperative days and/or after the chest tube removal, with the six pulmonary zones being evaluated. The patients were placed in a supine position, with the transducer in a lateral position at the level of the midaxillary line. To assess the parenchyma, the authors divided each hemithorax into six standardized zones delimited anteriorly by the parasternal region, anterior axillary, and posterior axillary lines and posteriorly by the posterior axillary and paravertebral lines. Zones 1 and 2 represent the anterosuperior and anteroinferior areas, and Zones 3 and 4 represent the superolateral and inferolateral areas. The back evaluation is performed with the patient in lateral decubitus, and Zones 5 and 6 are analyzed (Fig. 1). These represent the posterosuperior and inferior areas.^13^ The identification of the pleural drain can be performed by following its acoustic shadow until it enters the pleural space.

The ultrasound device used was the SonoSite NanoMaxx Ultrasound System (REF P11111-40), containing a convex transducer with a frequency between 2.0‒5.0 MHz (REF P11878-10), and both B and M modes. This examination was performed by a physician, a member of the Thoracic Surgery team at HC-FMUSP, who underwent specific training in thoracic ultrasonography. The CXR was evaluated by another physician, also a member of the Thoracic Surgery team at HC-FMUSP. The physician who performed the TUSG did not have prior knowledge of the CXR result, in the same way, that the CXR evaluator did not know the TUSG report. In total, 50 TUSGs and 50 CXRs, in 21 patients were evaluated by those standards. Although the authors had previously predicted a total of 63 TUSGs and 63 CXR, 13 patients had their chest tube removed before the 3^rd^ post-operative day, resulting in the 3^rd^-day exams coinciding with the post-chest tube removal exams.

Outcomes evaluated were the identification by both methods of the following parameters: subcutaneous emphysema, pneumothorax, pleural effusion, pleural thickening, pulmonary consolidation, diaphragmatic elevation, chest tube position and whether the imaging method suggests safety in the removal of the chest tube. The results were tabulated and subjected to statistical analysis, comparing sensibility, specificity, and accuracy. The Kappa statistic was applied for comparison between whether or not the same alteration is identified in the TUSG and CXR.

The sample size was calculated using the G.Power Software version 3.1 (Heinrich-Heine-Universität Düsseldorf-Germany), based on the publication by Chiappetta et al.^5^ with 24 patients. Considering the outcome of the overlap between TUSG and CXR in 19 of the 24 cases, the authors estimated an effect of about 0.76, by which the software calculated for an alpha of 0.05 and a beta of 0.95, determining a total sample size of 21 cases.

## Results

Twenty-one patients were evaluated, achieving 50 lung Ultrasonography Examinations (TUSG) and 50 Chest Radiographs (CXR). The studied population consists mostly of males in their forties. The majority had no comorbidities. However, 33 % have comorbidities such as Diabetes Mellitus, Systemic Arterial Hypertension, Hyperthyroidism, and Lymphoma. Most patients were diagnosed with pleural empyema and subjected to decortications (62 %). The right side was more commonly afflicted. Out of 21, 14 had one or more complications in the postoperative period, and two died; one died due to progression from a previously diagnosed lymphoma and another from sepsis after surgical complications. The characteristics of the study group, as well as the main findings in each exam, are described in Table 1.

**Table 1:** Characteristics of the study group.

Variables	n (%)^a^
**Sex**	
Female	8 (38.1)
Male	13 (61.9)
**Age, mean (Min‒Max)**	41.1 (16‒67)
**Comorbidities, n (%)**	
Yes	7 (33.3)
No	14 (66.7)
**Diagnosis, n (%)**	
Pulmonary Abscess	1 (4.7)
Fungal Infection	3 (14.3)
Bronquiectasis	3 (14.3)
Pleural empyema	10 (47.6)
Tuberculosis empyema	2 (9.5)
Lymphangioleiomiomatosis	1 (4.7)
Necrotizing pneumonia	1 (4.7)
**Type of surgery performed, n (%)**	
Decortication	13 (61.9)
Pulmonary resection	8 (38.1)
**Side of surgery, n (%)**	
Right	12 (57.1)
Left	9 (42.9)
**Complications, n (%)**	
No	7 (33.3)
Prolonged airway fistula	6 (28.6)
Reoperation	2 (9.5)
Renal failure	3 (14.3)
Subcutaneous emphysema	1 (4.7)
Heamothorax	1 (4.7)
Pleural effusion	1 (4.7)
Disease progression	1 (4.7)
**Duration of pleural drain in days, mean (Min‒Max)**	9.67 (1‒63)
**Duration of hospitalization in days, mean (Min**‒**Max)**	11.33 (3‒63)
**Evolution, n (%)**	
Discharged without pleural drain	13 (61.9)
Discharged with pleural drain	6 (28.6)
Death	2 (9.5)
**TUSG findings, n ( %)**	
Normal	0 (0)
Drain position normal	31 (64)
Drain position abnormal	0 (0)
No drain	11 (22)
Drain not identified	8 (16)
Subcutaneous emphysema	19 (38)
Pneumothorax or residual cavity	19 (38)
Pleural effusion	31 (62)
Pleural thickening	42 (84)
Pleural consolidation or atelectasis	37 (74)
Diaphragm elevation	4 (8)
**TUSG allows for drain removal, n (%)**	
Yes	15 (30)
No	24 (48)
No drain	11 (22)
**CXR findings, n (%)**	
Normal	1 (2)
Drain position normal	37 (74)
Drain position abnormal	2 (4)
No drain	11 (22)
Drain not identified	0 (0)
Subcutaneous emphysema	17 (34)
Pneumothorax or residual cavity	32 (64)
Pleural effusion	33 (66)
Pleural thickening	23 (46)
Pleural consolidation or atelectasis	41 (82)
Diaphragm elevation	2 (4)
**CXR allows for drain removal, n (%)**	
Yes	17 (34)
No	22 (44)
No drain	11 (22)

a Unless otherwise specified. TUSG, Lung Ultrasonography; CXR, Chest Radiography.

Both exams demonstrated similar results regarding their ability to safely predict the adequate moment for chest drain removal. TUSG suggested chest drain removal in 30 % of cases and CXR in 34 %. It is important to note that clinical parameters such as the volume of effusion being drained, and the presence of air leaks were also considered when deciding to remove a chest drain.

The authors performed statistical analysis (Table 2) to compare the TUSG sensitivity, specificity, positive predictive value, negative predictive value, precision, and accuracy to the CXR. It is noticeable that both exams have similar capabilities in detecting postoperative changes in the pleural space. However, the authors report that TUSG is statistically more accurate in detecting subcutaneous emphysema than CXR (*p* = 0.037). The analysis of other parameters showed no statistical difference.

**Table 2:** Statistical analysis comparing TUSG to CXR.

	Sensitivity	Specificity	Pos Pred Value	Neg Pred Value	Precision	Recall	Balanced Accuracy	Accuracy	p-value	Kappa
**Subcutaneous emphysema**	72.73 %	58.82 %	77.42 %	52.63 %	77.42 %	72.73 %	65.78 %	68.00 %	0.037	0.3068
**Pneumothorax and residual cavity**	72.22 %	43.75 %	41.94 %	73.68 %	41.94 %	72.22 %	57.99 %	54.00 %	0.366	0.1379
**Pleural effusion**	41.18 %	63.64 %	36.84 %	67.74 %	36.84 %	41.18 %	52.41 %	56.00 %	0.767	0.0468
**Pleural thickening**	18.52 %	86.96 %	62.50 %	47.62 %	62.50 %	18.52 %	52.74 %	50.00 %	0.711	0.0516
**Lung consolidation and atelectasis**	34.38 %	55.56 %	57.89 %	32.26 %	57.89 %	34.38 %	44.97 %	42.00 %	0.552	-0.0870
**Diaphragmatic elevation**	91.67 %	0.00 %	95.65 %	0.00 %	95.65 %	91.67 %	45.83 %	88.00 %	1.000	-0.0563

TUSG, Lung Ultrasonography; CXR, Chest Radiography.

The authors also applied the Kappa statistic to evaluate if the agreement between both exams would be better than chance alone.^14^, ^15^, ^16^ It is noticeable that there was no agreement better than chance in the evaluation of pleural effusion, pleural thickening, lung consolidation and atelectasis, and diaphragmatic elevation. However, there is a poor agreement in evaluating pneumothorax and residual cavities and a fair agreement in evaluating subcutaneous emphysema (Table 2).

## Discussion

In the past years, thoracic surgeons have expressed a renewed interest in TUSG as a mean of reducing patient exposure to radiation and obtaining images in real time that allows for fast and accurate diagnosis. Thoracic ultrasonography is shown to meet those criteria. Despite this, its use is still limited in the postoperative follow-up of thoracic surgery, with chest radiography being the initial examination and chest computed tomography the gold standard for diagnosing complications.^2^

Modern ultrasound devices feature several transducers and modalities. For thoracic evaluation, the use of a high-frequency linear transducer (5.0‒14.0 MHz) is indicated when a better analysis of the pleural line is desired, as well as for the detection of the transition point between the presence and absence of pleural slip (lung point) to accurately diagnosis pneumothorax.^1^ Both B-mode and M-mode can be used. In these cases, with the patient in the supine position, the transducer is positioned on the anterior chest wall, at its highest point, and in the lateral position, at the level of the midaxillary line.^1^ The low-frequency (1.0‒6.0 MHz) convex transducer, in B-mode, is indicated for examining pleural effusions and parenchyma. To visualize the effusions, the patient should be placed in a supine position and the transducer in a lateral position at the level of the midaxillary line.^1^ To assess the parenchyma, the division of each hemithorax into six zones is standardized, which are delimited anteriorly by the parasternal region, anterior axillary, and posterior axillary lines and posteriorly by the posterior axillary and paravertebral lines. Zones 1 and 2 represent the anterosuperior and anteroinferior areas and Zones 3 and 4 represent the superolateral and inferolateral areas. The back evaluation is performed with the patient in lateral decubitus, and Zones 5 and 6 are analyzed (Fig. 1). These represent the posterosuperior and inferior areas. The identification of the pleural drain can be done using both transducers.^17^^,^^18^

Goudie et al. (2012) presented that the TUSG is not accurate enough to replace the CXR. However, it can reduce the amount of radiation to which a patient can be subjected during hospitalization. In this study, which has a larger sample than the others, it was found that the use of TUSG has a sensitivity of 21.1 % and specificity of 94.7 % for the diagnosis of pneumothorax, and a sensitivity of 83.1 % and specificity of 59.3 % for detection of pleural effusions.^19^ However, Patella et al. (2018) and Chiappetta et al. (2018) concluded that TUSG could be used as an initial examination for postoperative follow-up of chest resections and replace chest radiography.^5^^,^^10^^,^^11^ With modern equipment and experienced hands, the sensitivity and specificity of TUSG for detecting pneumothorax were 86 %‒97 % and 97 %‒100 %, respectively. For pleural effusions, the sensitivity and specificity reach 100 %.^1^ Interestingly, the present data indicate a TSUG sensitivity and specificity, respectively, in relation to CRX, of 72.22 % and 43.75 % for pneumothorax and 41.18 % and 63.64 % for pleural effusions.

Papers published by Goudie et al. (2012) and Galetin et al. (2020) advocated that the use of TUSG should be reserved for selected cases due to the sonograph's low accuracy in detecting pneumothorax.^19^^,^^20^ However, a 2021 study by Galetin et al. that evaluated 208 patients concluded that conditions usually considered unfavorable for lung ultrasound did not impair the sensitivity or specificity of lung ultrasound.^21^ Recently, Malik et al. (2020) published a series of 297 patients subjected to various thoracic surgical treatments and concluded that the good use of TUSG may reduce the use of CXR in 61.6 % when searching for pleural effusion and pneumothorax.^21^ Patella et al. (2018) suggest that up to 80 % of CXR after chest tube removal can be avoided in the postoperative period of lung resection due to the high sensitivity of TUSG in confirming lung expansion and detecting pneumothorax.^10^ Chiappeta et al. (2018) emphasize that TUSG can be used in all phases of postoperative follow-up, with CXR being reserved for cases in which TUSG was doubtful or has some confounding factor, such as subcutaneous emphysema.^5^ Both authors conclude that TUSG is effective for detecting complications in the postoperative period of pulmonary resection.^5^^,^^10^ Cerfolio and Bryant (2011) reported an advantage in performing daily CXR in hypoxemic patients.^3^ That finding is in accordance with the present results, which suggest a similar sensitivity and specificity between imaging methods, allowing for the replacement of one, or the use of both, as complimentary exams, without prejudice to the patient. Noticebly, Grapatsas et al. goes even further, advocating that TUSG could, in fact, replace CXR in thoracic surgery patients.^22^

The Kappa statistic for inter-rater reliability for categorical variables was applied to detect differences in the assessment of both examiners (the one performing the TUSG and the one reviewing the CXR) to determine whether the investigated criteria occurred in both exams.^15^, ^16^ It is noticeable that there was no agreement better than chance in the evaluation of pleural effusion, pleural thickening, lung consolidation and atelectasis and diaphragmatic elevation. However, there is a poor agreement in evaluating pneumothorax and residual cavities and a fair agreement in evaluating subcutaneous emphysema.

None of those studies had evaluated whether TUSG is effective in diagnosing complications after surgical approach for infectious and inflammatory diseases. The authors have a specific concern in this subset of patients because of the higher frequency of pleural complications (10.86 %), mainly empyema and prolonged air fistula, as demonstrated in a previous paper.^12^ The authors hypothesized that if we had a better tool to conduct the postoperative period, perhaps we could quickly act and prevent some of the complications.

The present data is in accordance with the medical literature by showing similar sensitivity and specificity to CXR. It is important to notice that the authors compared the TUSG to CXR and not CCT. Even though the CCT is the gold standard for chest evaluation, in the daily practice, performing daily CT scans in patients represents unnecessary exposure to radiation and increased costs to the health care system. With this in mind, the authors sought to use the TUSG as a secondary means of postoperative evaluation on those patients, the primary being the CXR. Hence, the present data indicate that both TUSG and CXR provide the assistant physician with complementary information that can assist in patient decision-making. Therefore, the authors cannot affirm that one exam should be used in place of another, nor do we recommend changing our institution's protocol in utilizing CXR as the primary exam in the post-operative follow-up of cases. Nevertheless, the significant difference of this paper is its exclusive view of patients treated for infectious and inflammatory thoracic diseases, who can have more significant alterations in the pleural cavity.

The present study isn't without limitations. The small sample does not provide the generalizability desired in such a study. It is important to note that information bias may have been present. For example, when performing a TUSG in an intensive care unit (ICU) setting, the patient is obviously in a worse clinical state than a patient not in the ICU, which could induce some findings in the TUSG. That was completely avoidable when evaluating the CXR, due to the fact that this process could be done remotely. The second limitation of this paper can be the comparison of TUSG with CXR and not with CCT, which is considered the gold standard in pleural and pulmonary alterations detection. However, this was intentional since, for the reasons previously described of high cost and radiation exposure, the authors considered that a study comparing TUSG and CCT could not only raise critical ethical concerns but also be too far away from current clinical practice. With this in mind, the authors designed a more practical study, aiming to provide data that will be applicable on a routine basis. Finally, there is a need for a trained physician to perform the TUSG exams, however, the authors believe that once trained, those skills are easily applied in clinical scenarios.

The authors conclude that TUSG, in trained hands, is an important tool for thoracic surgeons in searching for postoperative complications regarding the surgical treatment of infectious and inflammatory thoracic diseases, and can be used as a complement, and not a substitute, to CXR when CCT is not feasible, or a more urgent diagnosis is needed. However, more studies are necessary to provide more accurate data regarding the use of TUSG after thoracic surgery for the treatment of inflammatory and infectious diseases.

## Declaration of competing interest

The authors declare no conflicts of interest.
